# Perceptions of Social Determinants of Successful Aging Among Older Men Living With HIV

**DOI:** 10.1093/geroni/igab046.1637

**Published:** 2021-12-17

**Authors:** Anna Rubtsova, David Moore, Dilip Jeste, Marcia Holstad

**Affiliations:** 1 Emory University Rollins School of Public Health, Emory University, Georgia, United States; 2 University of California, San Diego, San Diego, California, United States; 3 University of California San Diego, La Jolla, California, United States; 4 Emory University, Atlanta, Georgia, United States

## Abstract

The overall purpose of this qualitative study was to examine barriers and facilitators of successful aging among older men living with HIV (OMLH). Participants were recruited through HIV Neurobehavioral Research Program at the University of California, San Diego. Our sample included 14 OMLH: average age - 62 years old (range: 53 to 72), 79% white, 43% living alone, 79% men who have sex with men, 57% having college education or higher. Semi-structured interviews lasted from 43 to 114 minutes and were fully transcribed. Several themes emerged related to perceived barriers to successful aging stemming from social institutions: i.e., age discrimination and ageism, sexual and HIV-related stigma, social isolation, lack of resources, and food insecurity. Perceived institutional solutions promoting successful aging included mixed-age/inter-generational support groups, computer literacy training, health education, information and resources related to healthy lifestyle on a limited budget, and increased transparency of resources available to older adults.

